# Occipital lobe seizures and subcortical T2 and T2* hypointensity associated with nonketotic hyperglycemia: a case report

**DOI:** 10.1186/s13256-016-1010-8

**Published:** 2016-08-12

**Authors:** Fuyuko Sasaki, Sumihiro Kawajiri, Sho Nakajima, Ai Yamaguchi, Yuji Tomizawa, Kazuyuki Noda, Nobutaka Hattori, Yasuyuki Okuma

**Affiliations:** 1 Department of Neurology, Juntendo University Shizuoka Hospital, 1129 Nagaoka, Izunokuni-shi, Shizuoka 410-2295 Japan; 2 Department of Neurology, Juntendo University School of Medicine, 2-1-1 Hongo, Bunkyo-ku, Tokyo, 113-8421 Japan; 3 Institute of Oriental Medicine, Tokyo Women’s Medical University, 1-21-8 Tabata, Kita-ku, Tokyo, 114-0014 Japan

**Keywords:** Case report, Iron accumulation, NKH, Seizure, Subcortical T2 hypointensity, Subcortical T2* hypointensity

## Abstract

**Background:**

Nonketotic hyperglycemia often causes seizures. Recently, seizures associated with nonketotic hyperglycemia have been found to be associated with subcortical T2 hypointensity on magnetic resonance imaging, especially in the occipital lobes. However, the mechanism remains unclear, although iron accumulation is suggested. We present a case of occipital lobe seizures associated with nonketotic hyperglycemia supporting the hypothesis that the mechanism of subcortical T2 hypointensity is iron accumulation using gradient-echo T2*-weighted magnetic resonance imaging.

**Case presentation:**

A 65-year-old Japanese man complained of intermittent pastel-colored flashing lights. On neurological examination, he also had lower right-side quadrant hemianopia. No other abnormal neurological findings were found. On laboratory analysis, his blood glucose level was 370 mg/dL, HbA1c was 11.4 %, and serum osmolarity was 326 mOsm/L. No ketones were detected in urine. A magnetic resonance imaging scan of his head showed subcortical T2 and T2* hypointensity in his left occipital lobe. Single-photon emission computed tomography with I123-N-isopropyl-iodoamphetamine revealed hyperperfusion in the left dominant occipital lobe. These magnetic resonance imaging abnormalities resolved during clinical recovery and treatment to control his blood sugar level. Therefore, a diagnosis of occipital lobe seizures associated with nonketotic hyperglycemia was made.

**Conclusions:**

To the best of our knowledge, this is the first case of occipital lobe seizures associated with nonketotic hyperglycemia supporting the role of iron accumulation as a mechanism for subcortical T2 hypointensity using T2*-magnetic resonance imaging.

## Background

Nonketotic hyperglycemia (NKH) is a clinical syndrome comprising hyperglycemia, serum hyperosmolality, and intracellular dehydration with little or no ketoacidosis. NKH has been associated with various neurological manifestations. Several cases of subcortical T2 hypointensity after seizures associated with NKH have been reported [1, 2]. Although iron accumulation as a result of damage to axonal transport is suggested, the mechanism remains unclear. Here, we describe the case of a 65-year-old man with subcortical T2 hypointensity in the occipital lobe after seizures associated with NKH. Our case supports the hypothesis that the mechanism of subcortical T2 hypointensity is iron accumulation, because gradient-echo T2*-weighted magnetic resonance imaging (T2*-MRI) also revealed hypointensity in the same region.

## Case presentation

A 65-year-old Japanese man who had a past medical history of chronic obstructive pulmonary disease and no remarkable family medical history reported seeing intermittent pastel-colored flashing lights in his lower right-side visual field. Two weeks later, the flashing lights increased in frequency and appeared every 15–30 minutes for approximately 3 minutes at each occurrence. Consequently, the patient visited our hospital.

**Fig. 1:**
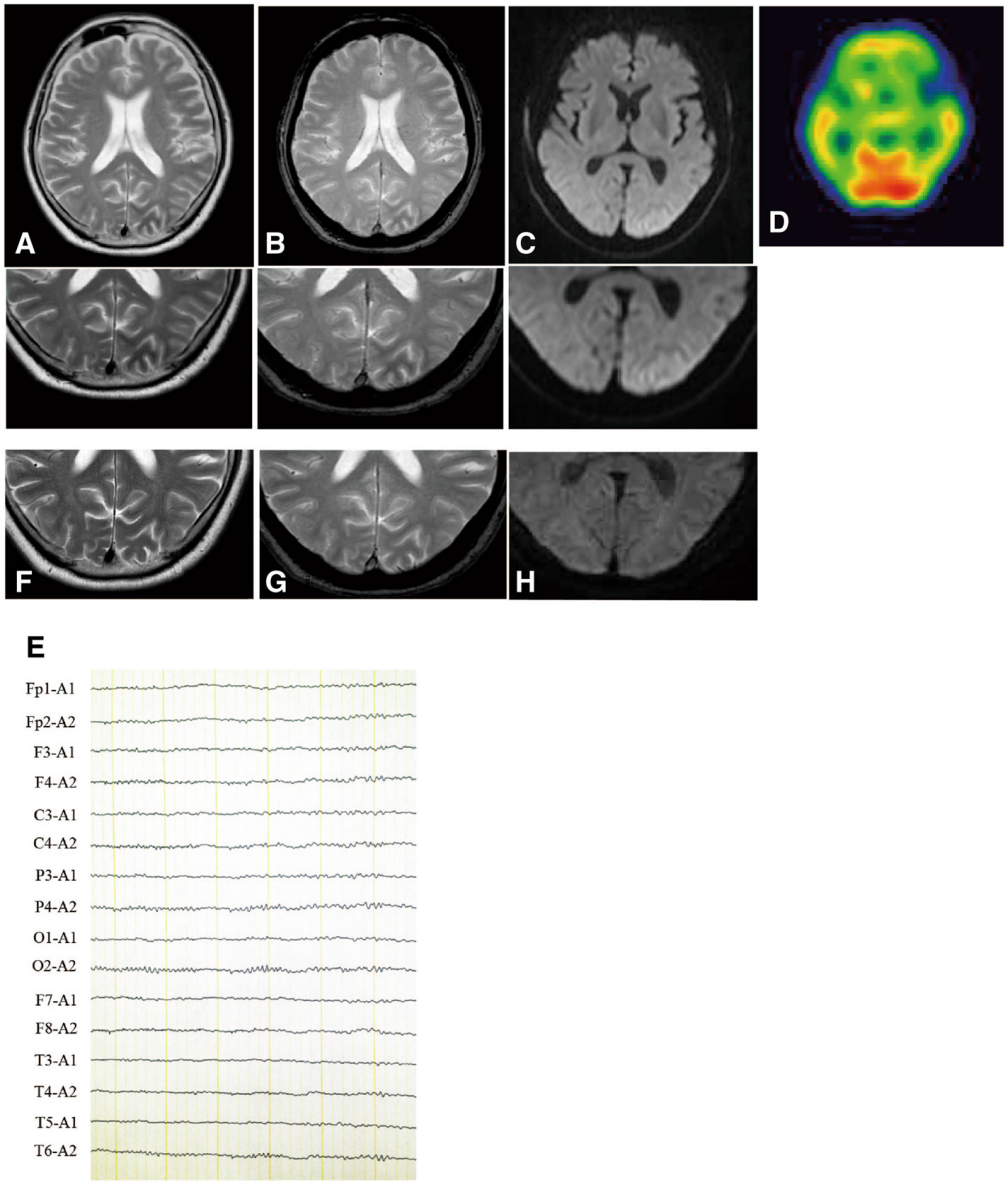
Magnetic resonance imaging on admission and 75 days after admission, single-photon emission computed tomography with I123-N-isopropyl-iodoamphetamine, and electroencephalography on admission. Axial T2-weighted (**a**) and T2* (**b**) magnetic resonance imaging on admission, demonstrating left occipital subcortical hypointensity. **c** Diffusion-weighted imaging on admission demonstrating slight cortical hyperintensity in the surrounding area of subcortical T2 hypointensity. Each lower panel is a higher magnification image of the occipital lobes. **f**, **g**, and **h** are higher magnification images of T2-weighted, T2*, and diffusion-weighted magnetic resonance imaging performed 75 days after admission, respectively, showing recovery. **d** Single-photon emission computed tomography with I123-N-isopropyl-iodoamphetamine demonstrated increased perfusion in the left dominant occipital lobe. **e** Electroencephalography demonstrated decreased alpha waves in the left occipital lobe

On neurological examination, in addition to intermittent flashing lights, our patient also had lower right-side quadrantanopsia. No other abnormal neurological findings were found. On physical examination, his height and body weight were 164.3 cm and 68.1 kg, respectively. He had no abnormal physical findings and his blood pressure, heart rate, and body temperature were 132/78 mmHg, 72/min, and 36.4 °C, respectively. On laboratory analysis, his blood glucose level was 370 mg/dL (normal value: 70–109 mg/dL), HbA1c was 11.4 % (normal value: 4.3–5.8 %), and serum osmolarity was 326 mOsm/L (normal value: 275–290 mOsm/L). No ketones were detected in his urine. A cerebrospinal fluid examination was within normal limits. A brain magnetic resonance imaging (MRI) scan demonstrated subcortical hypointensity in the left occipital lobe in gradient-echo T2-weighted MRI (T2-MRI) and T2*-MRI, and slight cortical hyperintensity in the adjacent area in diffusion-weighted imaging (DWI) (Fig. 1a–c). Gadolinium-enhanced T1-weighted MRI showed no enhancement of the areas. Single-photon emission computed tomography with I123-N-isopropyl-iodoamphetamine (IMP-SPECT) revealed hyperperfusion in the left dominant occipital lobe (Fig. 1d). Electroencephalography (EEG) was performed during intermittent periods, showing decreased alpha waves in the left occipital lobe, but no ictal discharge (Fig. 1e).

Consequently, we initially diagnosed NKH, although he had not been diagnosed with diabetes mellitus (DM), and considered that occipital lobe seizures were associated with NKH. Subsequently, we started intensive insulin therapy, vildagliptin (100 mg/day), and rehydration to control blood sugar. As his blood glucose became stable, his intermittent pastel-colored flashing lights lessened and disappeared 3 days after initiation of treatment. His quadrant hemianopia gradually improved, and completely resolved 2 weeks after treatment. A follow-up brain MRI scan, performed 75 days after the first MRI, showed disappearance of subcortical T2 and T2* hypointensity, and DWI cortical hyperintensity (Fig. 1f–h). His HbA1c improved to 6.6 % 3 months after initiation of treatment, and was stable for more than 2 years under treatment of vildagliptin (100 mg/day) and metformin (750 mg/day). He had no recurrence of intermittent pastel-colored flashing lights and quadrant hemianopsia for more than 2 years.

## Discussion

NKH is a serious acute complication of DM, typically in patients over 50 years with type 2 DM, and a clinical syndrome comprising severe hyperglycemia, hyperosmolarity, and intracellular dehydration occurring with little or no ketoacidosis. NKH has been associated with various neurological manifestations such as delirium, seizures, hemichorea-hemiballism, dysphagia, hemianopia, hemiparesis, and hemisensory loss. Most seizures associated with NKH are partial seizures with or without secondary generalization, which are refractory to antiepileptic drugs. Optimal treatment is correction of fluid and electrolyte abnormalities, and glycemic control. Particularly, the occipital lobe is potentially the most affected area, and occipital lobe seizures associated with NKH can cause flashing lights, well-formed visual hallucinations, photopsias, headache, vomiting, gaze and head deviation, blurry vision, and homonymous hemianopia. Seizures associated with NKH occasionally accompany characteristic MRI abnormalities, comprising subcortical T2 hypointensity in the white matter, hyperintensity of the overlying cortex, and focal overlying cortical contrast enhancement, reversible with the course of clinical recovery [1–3]. Despite markedly elevated HbA1c, the majority of cases, as well as our case, have only moderate hyperglycemia and absence of significant hyperosmolarity, and do not fulfill the diagnostic criteria for hyperosmolar hyperglycemic syndrome (HHS) (plasma glucose level of 600 mg/dL or greater, serum osmolality of 320 mOsm/kg or greater, profound dehydration, serum pH > 7.30, bicarbonate concentration > 15 mEq/L, small ketonuria, and absent-to-low ketonemia, and alterations in consciousness [4]). This suggests that long-standing hyperglycemia likely causes seizures rather than acute-onset extreme hyperglycemia [3].

Given hypointensity on T2-MRI and T2*-MRI, and occipital lobe lesion, we investigated possible microhemorrhage or posterior reversible encephalopathy syndrome (PRES). However, hypointensity on T2*-MRI in our case was not spotty, and the boundary was unclear. Blood pressure was not high, and PRES generally presents with T2 hyperintensity and does not present with subcortical T2 hypointensity. Therefore, we confirmed the diagnosis of occipital lobe seizure associated with NKH.

Despite increasing reports of similar cases, the detailed mechanism of T2 hypointensity remains uncertain. Neurons cannot exist without iron, which is an essential cofactor for enzymes involved with energy metabolism and synthesis of neurotransmitters. It is impossible to determine in our case and in similar cases whether excess iron caused neuronal cell damage or whether iron homeostasis was disrupted due to hyperglycemia. However, we speculate that accumulation of iron (enough to be reflected in T2-MRI and T2*-MRI) associated with disruption of axonal transportation, due to early cortical ischemia or excitotoxic damage from seizures, are potential mechanisms [2]. In our patient, MRI showed subcortical T2 hypointensity in his left occipital lobe in T2-MRI and T2*-MRI, strongly supporting the hypothesis that iron accumulation can contribute to these changes.

Ischemia or seizures without hyperglycemia can result in similar MRI abnormalities, indicating that subcortical T2 hypointensity is not specific to seizures associated with NKH [5]. However, many cases of seizure associated with NKH have been reported. Speculatively, cortical damage can be sufficient to lead to iron accumulation, and hyperglycemia can only result in acceleration of this pathophysiology. In contrast, many similar reported cases, as well as our case, are Asian. Thus, genetic or environmental factors may contribute to this pathophysiology.

## Conclusions

To the best of our knowledge, this is the first case of occipital lobe seizures associated with NKH supporting the role of iron accumulation as a mechanism for subcortical T2 hypointensity using T2*-MRI. However, the precise mechanism of iron accumulation remains uncertain. Further investigations are needed to reach verifiable conclusions.

## Abbreviations

DM, diabetes mellitus; DWI, diffusion-weighted imaging; EEG, electroencephalography; HHS, hyperosmolar hyperglycemic syndrome; IMP-SPECT, single-photon emission computed tomography with I123-N-isopropyl-iodoamphetamine; MRI, magnetic resonance imaging; NKH, nonketotic hyperglycemia; PRES, posterior reversible encephalopathy syndrome; T2*-MRI, gradient-echo T2*-weighted magnetic resonance imaging; T2-MRI, gradient-echo T2-weighted magnetic resonance imaging
